# Sleep Deprivation Impairs Consolidation of Cued Fear Memory in Rats

**DOI:** 10.1371/journal.pone.0047042

**Published:** 2012-10-17

**Authors:** Tankesh Kumar, Sushil K. Jha

**Affiliations:** School of Life Sciences, Jawaharlal Nehru University, New Delhi, India; University of Pittsburgh, United States of America

## Abstract

Post-learning sleep facilitates negative memory consolidation and also helps preserve it over several years. It is believed, therefore, that sleep deprivation may help prevent consolidation of fearful memory. Its effect, however, on consolidation of negative/frightening memories is not known. Cued fear-conditioning (CuFC) is a widely used model to understand the neural basis of negative memory associated with anxiety disorders. In this study, we first determined the suitable circadian timing for consolidation of CuFC memory and changes in sleep architecture after CuFC. Thereafter, we studied the effect of sleep deprivation on CuFC memory consolidation. Three sets of experiments were performed in male Wistar rat (n = 51). In experiment-I, animals were conditioned to cued-fear by presenting ten tone-shock paired stimuli during lights-on (7 AM) (n = 9) and lights-off (7 PM) (n = 9) periods. In experiment-II, animals were prepared for polysomnographic recording (n = 8) and changes in sleep architecture after CuFC was determined. Further in experiment-III, animals were cued fear-conditioned during the lights-off period and were randomly divided into four groups: Sleep-Deprived (SD) (n = 9), Non-Sleep Deprived (NSD) (n = 9), Stress Control (SC) (n = 9) and Tone Control (n = 7). Percent freezing amount, a hallmark of fear, was compared statistically in these groups. Rats trained during the lights-off period exhibited significantly more freezing compared to lights-on period. In CuFC trained animals, total sleep amount did not change, however, REM sleep decreased significantly. Further, out of total sleep time, animals spent proportionately more time in NREM sleep. Nevertheless, SD animals exhibited significantly less freezing compared to NSD and SC groups. These data suggest that sleep plays an important role in the consolidation of cued fear-conditioned memory.

## Introduction

Learning about fear could be an advantageous response mechanism but if its expression becomes too generalized, it leads to disorders associated with negative emotional memory. Fear conditioning is one of the widely used animal model paradigms for investigating the neural basis of negative memory [1], [2]. In cued fear-conditioning (CuFC), neutral conditioned stimulus (CS) (for example, light or tone) is paired with the fear inducing unconditioned stimulus (US) (such as foot-shock). Since both the stimuli are presented in a paired fashion during training, an emotionally neutral stimulus (tone/light) acquires fear inducing properties. The CS thus elicits responses like freezing, defecation, piloerection, which are otherwise characteristically generated by threatening stimuli [1], [3], [4]. Several studies suggest that in fear-conditioning, consolidation of emotion-event association is mediated through hippocampus-dependent but amygdala-dominant memory system [5], [6]. Animals trained for fear conditioning exhibit synaptic plasticity in the amygdala [7] and similarly, subjects with anxiety disorders also exhibit hyperactive amygdalar response to fearful stimuli [8]. Therefore, understanding about the impairment in consolidation of fearful memory may provide preventive clinical benefits in developing disorders associated with emotional memory.

It has been consistently observed in several studies that sleep deprivation impairs memory consolidation of a number of discrete learning tasks. For example, total sleep deprivation or insomnia impairs consolidation of declarative memory [9], motor adaptation task memory [10], motor sequence task memory [11], visual discrimination task memory [12] and trace-conditioned memory [13]; while selective REM sleep deprivation impairs memory consolidation of spatial learning (eight-box task) [14] and hidden-platform water maze learning [15]. It is not known, however, if sleep loss impairs the consolidation of negative memories too. There are evidences suggesting that sleep potentially facilitates consolidation of fearful memory and associated neuronal plasticity inside and outside of the amygdala [16], [17], [18], [19], [20]. For example, recently it has been reported that a night of sleep is sufficient to evoke qualitative changes in the emotional memory retrieval network and helps in shifting memory neural network from more diffused to a refined network into the amygdala and pre-frontal cortex [21]. The oscillations of theta activity, which help encode cued fear memories, increase its coherence in the amygdala and hippocampus after conditioning [22], [23] and these changes occur specifically during REM sleep [24]. It has also been reported that reactivation of neural network of episodic memories with cue helps reinforce memory retention, mainly during slow wave sleep [16]. These studies accentuate the importance of sleep in its highest strength for reinforcing retention of negative memories. Many of the symptoms of post-traumatic stress disorder (PTSD) (such as flashing, nightmare, hyper-arousal reactions etc.,) are developed due to over consolidation of negative memories [25]. It is known that sleep immediately after learning keeps emotional learning alive for years [26] and therefore, it is likely that sleep soon after a traumatic event may contribute in developing these fear-conditioned symptoms.

Several other factors, including circadian phases, may also influence the magnitude of learning and memory recall [27]. For example, active avoidance learning is enhanced in rodents during their active phase, whereas passive avoidance learning is enhanced during their rest period [28], [29]. However, studies of time of day effect on fear-conditioned learning have reported inconsistent results. While one study obtained no evidence for time-stamp effect on conditioned learning [30], another suggested that the circadian phase modulates consolidation of contextual fear memory but not CuFC memory [31]. It was subsequently reported that circadian phases do modulate both cued and contextual fear memory [32]. Furthermore, recently, it has been observed that a clock gene “*per-2*” in the hippocampus modulates trace fear-conditioning [33]. Therefore, the influence of time of day on consolidation of fear memory still remains largely undetermined.

In this study, we investigated the time of day effect and changes in sleep architecture after CuFC. More importantly, we also studied the impact of total sleep loss on consolidation of cued fear-conditioned memory in rats. We observed that rats trained for cued fear during the lights-off phase exhibited significantly more conditioning response. In addition, we observed that total sleep amount did not change after CuFC, but out of total sleep time, rats spent significantly more time in NREM sleep. Further, we found that total sleep deprivation immediately after training impaired the consolidation of fearful memory compared to non-sleep deprived and stress control animals.

## Methods

Male Wistar rats (250–300 gm) (n = 51) were used in this study. Animals were obtained from the university’s animal house facility and brought to the school’s animal room facility a week before starting the experiments. They were maintained on a 12∶12 light–dark (L:D) cycle (lights on at 7∶00 AM) at 24°C room temperature in the animal room, and food and water were given *ad lib.* All procedures used in this study were approved by the Institution’s Animal Ethical Committee (IAEC) of Jawaharlal Nehru University, New Delhi, India.

We performed three sets of experiments. Experiment-I: to study time of day effect on consolidation of CuFC memory; Experiment-II: to study the changes in sleep architecture after CuFC training and testing; and Experiment-III: to study the effect of sleep deprivation on consolidation of CuFC memory.

### Experiment-I: Fear-conditioning During Lights-on and Lights-off Phases

#### Procedure for cued fear-conditioned training and testing

**Figure 1:**
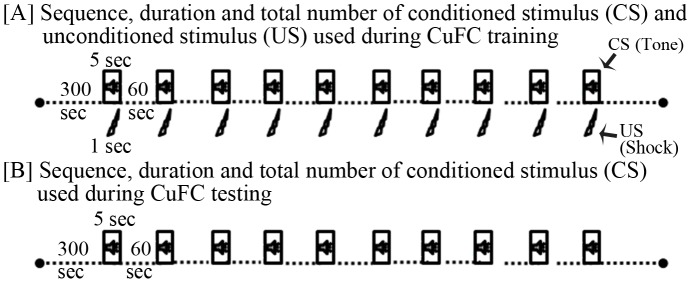
Cued fear-conditioning protocol. (A) Ten paired conditioned (tone: 2200 Hz, 90 dB) and unconditioned (foot-shock: 0.8 mA AC current) stimuli were presented over a period of 1200 sec for fear conditioning. Tones were presented at an interval of 60 sec for 5 sec which co-terminated with 1 sec foot-shock. (B) Animals were tested for cued fear-conditioning the next day, during which only conditioned stimulus (tone) of similar sequence, duration and number (as used during cued fear-conditioned (CuFC) training) was presented.

Two different groups of rats were trained for cued fear-conditioning during lights-on (n = 9) and lights-off (n = 9) period and increase in percent freezing amount on the testing day compared to baseline days was accounted as an outcome measure of learning. We used auditory tone (2200 Hz, 90 dB, 5 sec duration) as CS and an electric footshock (0.8 mA AC current, 1 sec duration) as US. Fear-conditioned training was performed in a shock chamber (Coulbourn Inc, USA), while the conditioned response was measured in a neutral chamber. The shock chamber was kept inside a sound and light dampened box (26″×24″×18″ black box) to minimize external disturbances during experiments. Before commencing the fear-conditioning training, animals of lights-on and lights-off groups were habituated in a neutral chamber for four days during 11∶00 AM–11∶30 AM and 7∶00 PM–7∶30 PM, respectively. Baseline freezing was recorded on two consecutive days (Days 1 and 2) in a computer through Freeze Frame software (Coulbourn Inc, USA) using CCTV camera (Panasonic WV-BP 334). On Day 3, rats in the lights-on group were trained for cued fear-conditioning in the shock chamber at 11∶00 AM and rats in the lights-off group were trained at 7∶00 PM. CuFC training was performed in a different room by a different person, who had not handled the animal before. The new person brought the animal through a different route to the training room to avoid contextual reminders. The animal was immediately placed in the shock chamber and conditioning stimuli were presented through a computer controlled protocol (using Freeze Frame software). Tone-shock stimuli were presented over a 10 min time period in ten sessions. The first tone was delivered with a delay of 300 sec while the subsequent nine tones were delivered at an interval of 60 sec. In every session, tone always co-terminated with an electric foot-shock (delivered through the grid floor) (Figure 1A). Animals’ behavior was captured continuously through a camera for 20 mins (time matched hour during baseline days: 5 mins prior to the tone, 10 mins during and 5 mins after the tone presentation) for offline analysis of freezing response. On the testing day (Day 4) (24 hours later), rats were re-exposed to CS alone in the neutral chamber. Tone was presented on post-conditioning day in a similar fashion as it was presented during the training period but without foot-shock (tones were presented in 10 sessions; 5 mins after keeping the animal in the chamber) (Figure 1B). Freezing response was recorded (time matched hour during baseline and training days) and was analyzed offline.

The data of animals trained for CuFC task during the lights-off phase in experiment-I (n = 9), was also used in non-sleep-deprived (NSD) group under experiment-III. Since the protocol in these two groups was precisely similar, therefore, we intended to avoid experimental repetition and unnecessary use of animals.

### Experiment-II: Changes in Sleep Architecture after CuFC Training and Testing

#### Surgical procedures for polysomnographic recordings

A total of eight animals were prepared for chronic sleep-wakefulness (S-W) recording using similar procedure as has been reported earlier [34]. Briefly, under inhalation anesthesia (isoflurane) and sterile conditions, animals were prepared for polysomnographic recordings [34]. After anesthetizing the animal, scalp was shaved and head was fixed in the stereotaxic instrument. A midline incision was made and skin was cut aside to expose the skull for electrode implantations. Two pairs of small, stainless-steel screw electrodes were affixed on the skull above the frontal and parietal cortices to record electroencephalogram (EEG). Three electrodes (flexible insulated wires except at the tip) were implanted in the dorsal neck muscles to record bipolar electromyogram (EMG) (third EMG was implanted as a safeguard). One screw electrode was fixed lateral to the midline in the nasal bone as a reference. Free ends of EEG, EMG and reference electrodes were connected to a 9-pin miniature connector, which was cemented onto the skull with dental acrylic and finally, the neck skin was sutured. Animals were treated post-operatively with dexamethasone to reduce brain inflammation and nebaself powder (antibiotic) to control infection.

#### Polysomnographic recording and data analysis

Sleep architecture was recorded for six hours (11∶30 AM–5∶30 PM) during the lights-on phase soon after CuFC training and testing. After recovery from surgery, animals were initially habituated in the recording chamber for four consecutive days. On subsequent two days, baseline S-W was recorded in a computer through *Somnologica Science software* and *Embla A10* (Medcare Flaga, Iceland). EEG signals were processed with a high-pass 0.1 Hz and low pass 40 Hz filters, while EMG was processed with high pass 10 Hz and low-pass 90 Hz filters at 100 Hz sampling rate. The recordings were saved for off-line analysis.

Offline, polysomnographic records were displayed in *Somnologica* and were manually scored using 4-sec epochs for six hours period from 11∶30 AM–5∶30 PM employing the standard criteria for rats. Low voltage and high frequency EEG waves associated with increased motor activity were analysed as wake; high voltage, low frequency EEG waves (0.5–4 Hz) and decreased motor activity were analysed as NREM sleep and low voltage, high frequency EEG waves with a prominent theta peak (5–9 Hz) and nuchal muscle atonia were analysed as REM sleep. The total time spent in wake, NREM and REM sleep were calculated. These values were expressed as hourly and total mean percent of the total recording time. Further, we also characterized percent NREM sleep and REM sleep out of the total sleep time. The differences in the vigilant states after CuFC training and testing were compared statistically with baseline day (one-way ANOVA followed by tukey posthoc test).

### Experiment-III: Effects of Sleep Deprivation on Consolidation of CuFC Memory

#### Fear conditioning in sleep deprived (SD), non-sleep-deprived (NSD) stress control (SC) and tone control (TC) groups during the lights-off phase

From Experiment-I, we determined that animals trained for cued fear-conditioning during the lights-off phase exhibited significantly better conditioning response. Therefore, further experiments were performed during this phase (starting from 7∶00 PM). To study the role of sleep in consolidation of CuFC memory, animals were further randomly divided into four groups: (i) Sleep-Deprived (SD) group (n = 9); (ii) Non-SD (NSD) group (n = 9) (iii) Stress Control (SC) group (n = 9) and (iv) Tone Control group (n = 7). Animals in SD group were total sleep deprived continuously for 6 hours soon after training. Six hour period was chosen because several reports suggest that initial six hour period soon after training is crucial for memory consolidation [13], [17], [35], [36]. Sleep deprivation was performed using three different approaches in all animals: (a) gentle handling, (b) tactile stimulation and (c) using a rotating disk. The experimenter kept the animal awake for initial approx. 2 hours with gentle handling and tactile stimulation, but with time it became quite difficult to keep the animal awake, hence, the rotating disk was used. In the rotating disk method, a vertically fixed barrier [a wall made of transparent plexiglass plate (8″ X 8″)] extending from the sidewall to the center of the cage and barely few mm up from the rotating disk prevented the animal to sleep, as the laid-back animal would bump into the barrier and thus forced to remain awake. The experimenter continuously observed the animal during the entire sleep deprivation period. By using a combination of these approaches, we have found previously that animals were 98% awake during the sleep deprivation period [35], [36]. Mild drowsiness (2%) did not alter the gross changes in sleep-dependent synaptic plasticity. After six hours of sleep deprivation, SD rats were immediately brought back to the animal colony and left undisturbed. Rats in NSD group (*the same animals trained during the lights-off phase in Experiment-I*) were kept in the animal room soon after training. In stress control group, we used bright light (400 Lux) (continuously for six hours with time matched hours of sleep deprivation), which induces robust stress and anxiety in the rat [37]. The aversive condition as such does not impair memory consolidation [38], however, acute alteration in circadian phase (6 hours phase delay by keeping the animal for extended hours in light) causes marginal impairment in the consolidation of fear-conditioned memory [39]. Also, changes in light intensity alter S-W primarily during the lights-on phase and not during the lights-off phase [40]. For all these reasons, we used bright light protocol, a reasonably apt stress control, for corresponding 6 hours sleep deprivation. Using similar conditioning protocol, animals in SC group (n = 9) were fear-conditioned and soon after kept in the stress chamber for next 6 hours under intense light. Finally, in the tone control group (n = 7), we presented only tone (2200 Hz, 90 dB) during training (Day 3). This control experiment was performed to ascertain that the induced freezing on post-conditioning day was indeed a condition mediated response and not attributable to tones. Animals in all four groups were re-exposed to CS (tone) alone 24 hours later at time matched hour of CuFC training during the lights-off phase. Tone induced conditioning effect (i.e. freezing response) was recorded on testing day as an outcome measure of fearful learning.

#### Analysis of freezing behavior

Freezing behavior was analyzed offline by using Freeze View software (Coulbourn Inc, USA). The percent freezing response before tone presentation (300 sec), during tone presentation (600 sec) and post tone presentation (300 sec) were calculated for baseline, CuFC training and testing days. Bout length of the motion index was kept at 5 sec which means that if the animal remained static for 5 sec and more, then only it was registered as freezing. Also, the freezing threshold was kept at 10%, at which the freezing bout peak falls around 25–75 sec motion index. The freezing response was thus analyzed by using computer software only, having a stringent criteria and no manual intervention. The average % freezing values were calculated at every 300 sec. We also calculated the cumulative freezing response on testing day for all groups. Changes in the freezing response in different groups were compared statistically using one-way ANOVA followed by tukey posthoc test.

## Results

### Experiment-I: Time of Day Effect on Cued Fear Learning

#### Changes in freezing response on post-conditioning day during lights-on and lights-off periods

**Figure 2:**
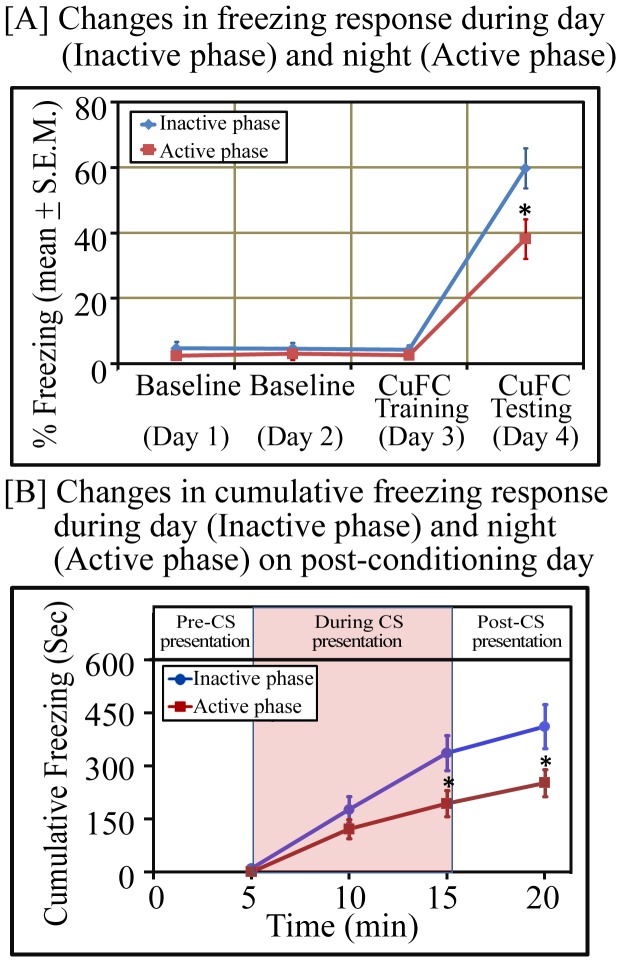
Percent and cumulative freezing response in cued fear-conditioned animals during lights-on and lights-off phases. Rats trained for CuFC exhibited significant increase in [A] percent freezing (p<0.05; F_(1,17)_ = 6.26) and [B] cumulative freezing response (p<0.05; F_(1,17)_ = 4.98 at the end of tone presentation and p<0.05; F_(1,17)_ = 4.75 after 5 mins of tone presentation) after tone presentation during lights-off phase compared to lights-on phase (one-way ANOVA). Data are expressed as [A] percent freezing time spent during and after tone presentation and [B] total time spent (sec) in freezing at every five mins before, during and after tone presentation. Abbreviation:- CS: conditioned stimulus. * signifies p<0.05.

With this experiment, we examined time of day effect on cued fear-conditioning. Rats trained for cued fear-conditioning both during lights-on (n = 9) and lights-off (n = 9) phases showed significant conditioning effect of tone during testing compared to baseline day (Figure 2A and B). The percent mean (± S.E.M.) freezing response on testing days during the lights-on and lights-off phases were 38.18±6.05 (p<0.001; F_(1, 17)_ = 31.29) and 59.84±6.19 (p<0.001; F_(1, 17)_ = 73.19), respectively. Animals in both groups thus exhibited significant freezing response on testing days; nevertheless, rats trained during the lights-off phase showed significantly more freezing response compared to rats trained during the lights-on phase (Figure 2A and B). Rats trained for cued fear during the lights-off phase exhibited 56.73% more freezing (p<0.05; F_(1, 17)_ = 6.26) compared to rats trained during the lights-on period (Figure 2A). However, there were no significant changes in freezing response between groups during baseline and CuFC training days. Additionally, temporal cumulative fear conditioning response at every 5 mins interval, before, during and after tone presentation on the post-conditioning day exhibited a successive increase in freezing response during each interval in both groups. Animals trained during the lights-off period, however showed significantly more freezing response after 10 mins (p<0.05; F_(1,17)_ = 4.98) and 15 mins (p<0.05; F_(1,17)_ = 4.75) of tone inception (Figure 2B). These results suggest that rats trained for cued fear-conditioning during the lights-off phase showed a significantly better learning compared to rats trained during the lights-on phase.

### Experiment-II: Changes in Sleep Architecture after CuFC Training and Testing

#### Changes in NREM sleep and REM sleep amount (out of total recording time and total sleep time)

**Figure 3:**
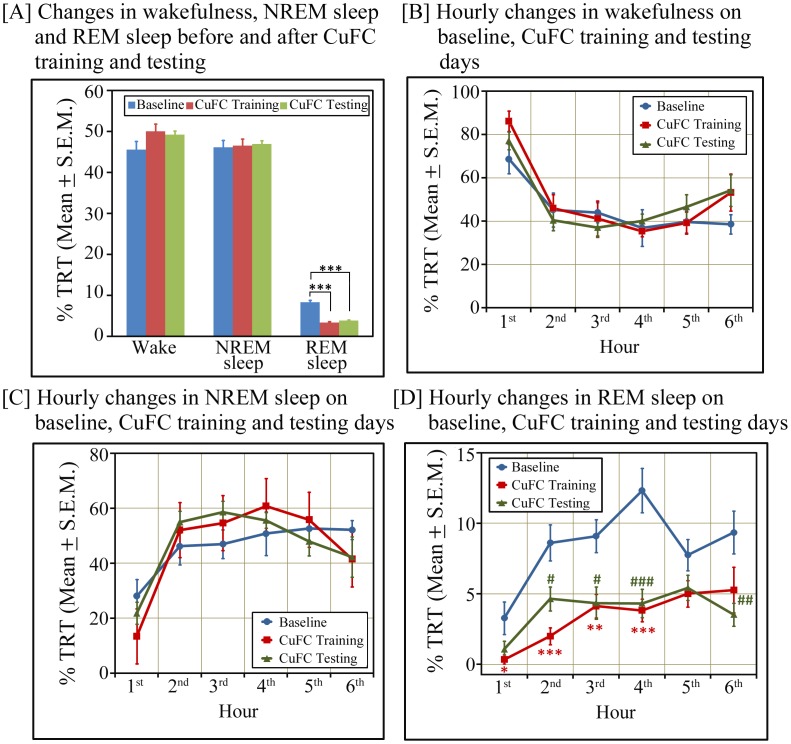
Changes in sleep-wake architecture (out of total recording time) after cued-fear conditioning during lights-on phase. [A] Percent changes in wakefulness, NREM sleep and REM sleep amount after CuFC training and testing compared to baseline day. Out of total recording time, % wakefulness and % NREM sleep amount on CuFC training and testing days did not change, however, % REM sleep amount significantly decreased on CuFC training (p<0.001; F_(1,15)_ = 90.69) and testing (p<0.001; F_(1,15)_ = 82.63) days compared to baseline day. Hourly percent time spent in [B] wakefulness [C] NREM sleep and [D] REM sleep during the lights-on phase. REM sleep amount significantly decreased on training day (except 5^th^ and 6^th^ hours) and testing day (except 5^th^ hour). * signifies changes in REM sleep amount on CuFC training day, while # signifies changes in REM sleep amount on CuFC testing day compared to the time matched hour on baseline day. Significance level: * =  p<0.05; ** =  p<0.01; *** =  p<0.001; #  =  p<0.05; ##  =  p<0.01; ###  =  p<0.001.

**Figure 4:**
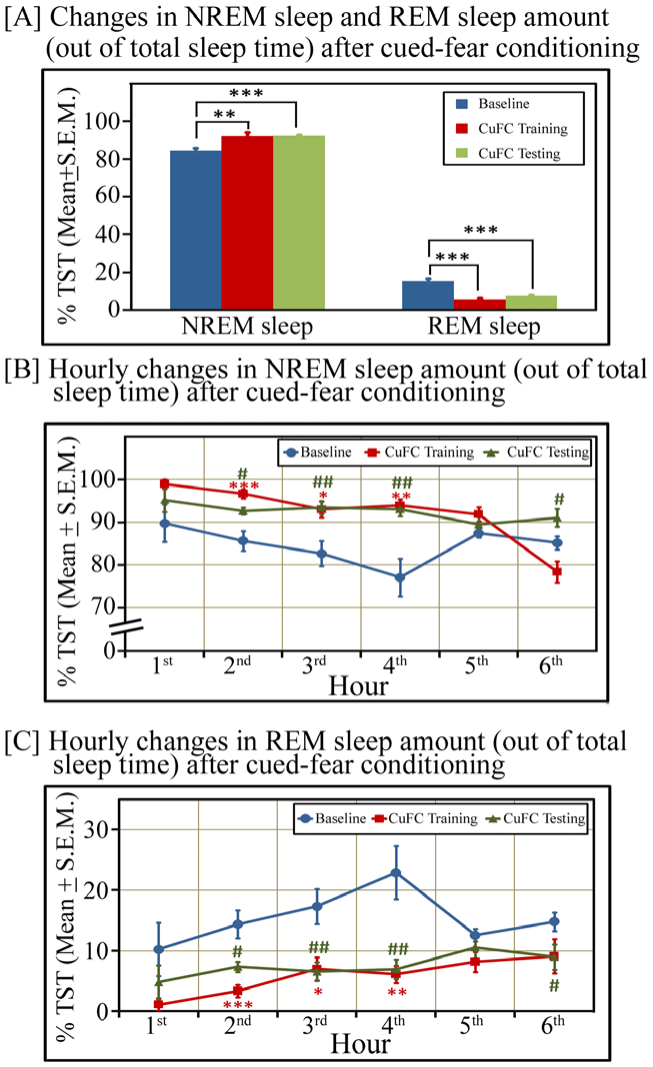
Changes in sleep architecture (out of total sleep time) after cued fear conditioning during the lights-on phase. [A] Percent changes in NREM sleep and REM sleep amount after CuFC training and testing compared to baseline day. Out of total sleep time, % NREM sleep amount significantly increased on CuFC training (p<0.01; F_(1,15)_ = 9.86) and testing (p<0.001; F_(1,15)_ = 31.94) days, while % REM sleep amount significantly decreased on CuFC training (p<0.001; F_(1,15)_ = 46.78) and testing (p<0.001; F_(1,15)_ = 31.94) days compared to baseline day. Hourly percent time (out of total sleep time) spent in [B] NREM sleep and [C] REM sleep. NREM sleep amount significantly increased on training day during 2^nd^, 3^rd^ and 4^th^ hours while it significantly increased during 2^nd^, 3^rd^, 4^th^ and 6^th^ hours on testing day. On the other hand, REM sleep amount significantly decreased on training day during 2^nd^, 3^rd^ and 4^th^ hours and during 2^nd^, 3^rd^, 4^th^ and 6^th^ hours on testing day. * signifies changes on CuFC training day, while # signifies changes on CuFC testing day compared to the time matched hour on baseline day. Significance level: * =  p<0.05; ** =  p<0.01; *** =  p<0.001; #  =  p<0.05; ##  =  p<0.01.

Post-learning sleep may play an important role in the consolidation of fear-conditioned memory; it was imperative, therefore, to know how much time animals spent in sleep after fear conditioning. We observed that out of total 6 hours recording period after CuFC training and testing, total wakefulness and NREM sleep amount did not change after CuFC training and testing compared to baseline (Figure 3A). However, REM sleep was significantly decreased on both days (Figure 3A). On conditioning day, REM sleep was 59.21% less (p<0.001; F_(1,15)_ = 90.69), while on post-conditioning day, it was 53.71% less (p<0.001; F_(1,15)_ = 82.63) (Figure 3A) compared to baseline day. Hourly distribution of wake, NREM sleep and REM sleep states on baseline, conditioning and post-conditioning days are shown in Figure 3B–D. Although, overall NREM sleep amount did not change, but NREM sleep amount was significantly high during a total of 3 hours period (between 2^nd^ hour - 4^th^ hour) [Baseline: 47.97±2.16; CuFC training: 55.82±1.72 (p<0.05; F_(1,15)_ = 8.10); CuFC testing: 56.41±2.29 (p<0.05; F_(1,15)_ = 7.19)]. On the contrary, overall REM sleep amount as well as REM sleep amount at every hour (except 5^th^ hour) was significantly less on CuFC training and CuFC testing days (Figure 3D). Total wakefulness and sleep amount was comparable on baseline, CuFC training and testing days; nevertheless, REM sleep was significantly decreased. It is, therefore, likely that the animal spent more time in NREM sleep. We observed that out of total sleep time, CuFC trained animals spent 8.83% more time in NREM sleep (p<0.01; F_(1,15)_ = 9.86) on conditioning day and 9.25% more time in NREM sleep (p<0.001; F_(1,15)_ = 31.94) on post-conditioning day compared to baseline day (Figure 4A). Hourly distribution of NREM sleep and REM sleep states (out of total sleep time) on baseline, conditioning and post-conditioning days are shown in Figure 4B–C. These results suggest that the sleep architecture changes after cued fear-conditioning and animals spent proportionately more time in NREM sleep soon after fear-conditioning.

### Experiment-III: Effects of Sleep Deprivation on Consolidation of Cued Fear-conditioned Memory

#### Changes in freezing response on post-conditioning day in sleep-deprived, non-sleep-deprived and stress control groups during the lights-off phase

**Figure 5:**
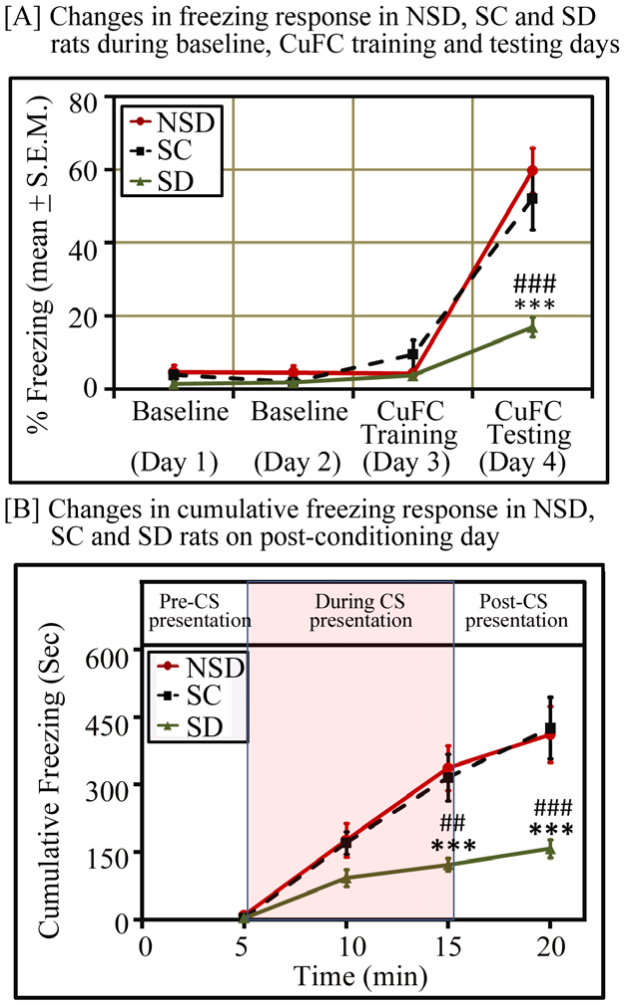
Percent and cumulative freezing responses in sleep-deprived (SD), non-sleep-deprived (NSD) and stress control (SC) rats. [A] On post-conditioning day, SD animals exhibited significantly less freezing compared to NSD and SC control animals (p<0.001 compared to NSD group and SC group) (one-way ANOVA). [B] Changes in cumulative freezing response in SD, NSD and SC animals. NSD and SC groups demonstrated a sequential increase in freezing response during each 5 mins interval; however, SD animals exhibited significantly less freezing at the end of conditioned stimulus (CS) presentation (p<0.001 compared to NSD animals; p<0.01 compared to SC animals) and also during the post-CS presentation (p<0.001 compared to both NSD and SC animals) (one-way ANOVA). Significance level: ***  =  p<0.001 (compared to NSD group); ##  =  p<0.01; ###  =  p<0.001 (compared to SC group).

Since a robust freezing response was observed during the lights-off phase, hence, the effect of sleep loss on conditioned freezing response was determined in rats trained for cued fear-conditioning during the night. A short term total sleep deprivation (6 hours) soon after CuFC training (Day 3) induced significantly less freezing response on post-conditioning day (Day 4) in SD animals compared to NSD and SC animals. The percent mean (± S.E.M.) conditioned freezing response in NSD and SC groups were 59.84±6.19 and 52.07±8.43, respectively. However, SD animals demonstrated significantly less freezing (17.01±2.71%) compared to NSD (p<0.001; F_(1,17)_ = 40.15) and SC (p<0.001; F_(1,17)_ = 15.67) groups during the post conditioning day (Day 4) (Figure 5A). Freezing response within groups was not significant on baseline and CuFC training days. Further, tone alone did not induce significant freezing response in animals of tone control group (Table 1), which attributed the induced freezing in other groups as an explicit conditioning effect.

**Table 1:** Changes in percent freezing response in the tone control group.

	Baseline (Day 1)	Baseline (Day 2)	CuFC Training (Day 3)	CuFC Testing (Day 4)
Mean (n = 7)	4.38	4.22	6.14	4.52
S.E.M.	2.17	1.78	1.83	2.04
p value		0.95	0.55	0.96
F value (1,13)		0.003	0.38	0.002

Additionally, temporal cumulative fear conditioning response was analyzed at every 5 mins interval, before, during and after tone presentation on the post-conditioning day (Figure 5B). It was observed that animals in the control groups (NSD and SC groups) exhibited a sequential increase in freezing response during each interval; however, the freezing response fell significantly flat after 10 mins of tone inception in SD group. The freezing response before and during the 5 min CS presentation were comparable in all groups but it was significantly less in SD group at the end of CS presentation (compared to NSD  =  p<0.001; F_(1,17)_ = 17.15; compared to SC  =  p<0.01; F_(1,17)_ = 13.23) and also during the post-CS presentation (compared to NSD  =  p<0.001; F_(1,17)_ = 15.09; compared to SC  =  p<0.001; F_(1,17)_ = 14.22) (Figure 5B).

## Discussion

Sleep soon after exposure to traumatic events may facilitate over-consolidation of negative memories [26]. It has been suggested, therefore, that sleep deprivation soon after life-threating events may help protect the subjects from developing anxiety disorders and associated symptoms such as hyper-arousal reaction, nightmare etc. [26], [41]. Cued fear-conditioning protocol is widely used in the animal model to understand the psycho-physiological alterations in anxiety disorders [1], [34], [42]. Using cued fear-conditioning protocol, here we show that six hours of total sleep deprivation soon after CuFC training causes memory deficit. The effects can exclusively be attributed to sleep deprivation, as SD animals exhibited significantly less freezing compared to NSD and SC rats trained during the same time period. Presenting stress to SC animals for a comparable sleep deprivation time period did not alter memory consolidation, suggesting that sleep plays an important role in the consolidation of negative memories. Further, cumulative freezing data shows that animals in all three groups responded to CS in a similar fashion during the initial 5 mins. SD rats, however, showed a dramatic reduction in freezing response compared to NSD and SC groups in the subsequent time period during which SD animals appeared oblivious to the CS. Our results thus suggest that sleep loss immediately after fear learning causes deficits in memory consolidation.

Earlier it has been reported that sleep deprivation selectively impairs memory consolidation for contextual fear-conditioning only but not CuFC memory [17], [43]. However, our results suggest that sleep deprivation does impair cued fear-conditioned memory. The difference in results of our study with those of earlier studies could be attributed to either or both of the two reasons: (i) we observed that CS induced comparable freezing response in NSD, SC and SD group of animals for the initial 5 mins (Figure 5B). However, freezing response in SD animals decreased significantly during the subsequent testing period. Other groups have assessed the freezing response to CS for 2–3 mins only since tone inception [17], [43]. As seen in our study, a significant decrease in the freezing response in SD animals occurred outside the 2–3 min window, hence they were probably unable to register the sleep deprivation induced altered freezing response. (ii) The other reason of varying results could be the effect of time of day during which the conditioning was done. We performed this study during the lights-off phase while other groups [17], [43] have performed the experiments during the lights-on phase, when animals exhibit less freezing anyway after cued fear-conditioning (Figure 2A and B). Hence, it is possible that in their studies the changes in freezing response might have not been large enough to be statistically significant.

Circadian phases modulate fear-conditioning [31], [33] and it has been reported that mice can acquire better conditioning to cued fear during the lights-on phase [32]. However, our results suggest a significant more robust cued fear-conditioning effect during the lights-off period and the contrary results may be attributed to species difference [39]. Circadian modulation results in augmented learning if the learning happens during the animal’s active phase as it has been demonstrated that nocturnal and diurnal species exhibit antithesis effect of time of day on encoding of memory information [44]. Our data are also consistent with this finding and suggest that the active phase of the circadian system in the rat is important for better conditioning to fearful memory.

Fear-conditioning may affect sleep-wakefulness. Several previous reports have demonstrated that REM sleep amount significantly decreased after CuFC but NREM sleep amount did not change [34], [45], [46]. Our results are also consistent with earlier findings. Since total sleep amount did not change after CuFC but REM sleep amount decreased, hence within sleep, NREM sleep proportion increased after CuFC. On the contrary, decrease in REM sleep after CuFC raises an intriguing question “*why does REM sleep decrease after CuFC? Does it play a role of a negative modulator in the consolidation of CuFC memory*?” Further study is needed to unravel the precise role of REM sleep in strengthening CuFC memory but currently it appears that NREM sleep plays an important role in the consolidation of CuFC memory as (i) its amount increased significantly during a three hours window after CuFC and (ii) within sleep, NREM sleep increased significantly.

Amygdalar neurons manifest necessary plastic changes with cued fear-conditioning [47], [48] and re-express physiological plasticity during sleep which was acquired during wakefulness, [19] suggesting that the sleeping brain reinforces neural encoding in the amygdala. Additionally, it has been shown that sleep deprivation alters synaptic plasticity and membrane excitability in the hippocampal neurons [20], [49], [50] and synaptic up-scaling in the cortical neurons [35], [36], [51]. If amygdalar neurons also exhibit such phenomenon with sleep deprivation is not known, but based on the facts that sleep deprivation alters synaptic plasticity in the hippocampal mediated contextual fear-conditioning circuitries, it can be argued that sleep loss may induce deficit in retention of fear memory trace after cued fear-conditioning. Similarly, the amygdala exhibits heightened response in PTSD patients during symptomatic states and the amygdala responsivity positively correlates with symptom severity in PTSD [52]. It is likely that sleep restriction soon after exposure to a traumatic incident would prevent induction of neuronal plasticity in the amygdala and hence, would help preclude the development of PTSD [26], [41].

In summary, our results show that sleep deprivation soon after CuFC training impairs cued fear memory. Sleep deprived animals exhibit reduced conditioned response to tone after 5 mins of its inception compared to control animals. Our study suggests that sleep plays an important role in the consolidation of cued fear memory and thus supports the view that sleep deprivation can possibly be used as a therapeutic measure to counteract the development of anxiety disorders such as PTSD in humans.
